# Improvement of binocular summation in intermittent exotropia following successful postoperative alignment

**DOI:** 10.1038/s41598-021-95049-9

**Published:** 2021-08-02

**Authors:** YuePing Li, Juan Ding, Wei Zhang

**Affiliations:** Tianjin Eye Hospital, Tianjin Key Laboratory of Ophthalmology and Vision Science, Affiliated Eye Hospital of NanKai University, Clinical College of Ophthalmology of Tianjin Medical University, Tianjin, 300020 China

**Keywords:** Eye diseases, Eye abnormalities, Ocular motility disorders, Vision disorders

## Abstract

To investigate the improvement of binocular summation (BiS) at high contrast (100%) and different low contrasts (10, 5 and 2.5%) in patients with intermittent exotropia (IXT) after successfully postoperative alignment. A total of 76 patients (aged 9–40 years) with IXT and poor control at distance before surgery were enrolled in this study. The postoperative deviations ranged between 4 PD esophoria to 10 PD exotropia in the primary position (at near and at distance) in all the enrolled patients. The follow-up visits were 2–3 months after the surgery. We analyzed preoperative and postoperative BiS and the proportions of patients with different BiS for the high contrast and the low contrasts. Binocular summation (BiS) was classified into three situations: binocular summation, equal and inbibition. The results of the distant random dots stereograph (RDS) were grouped into A, unable to recognize; B, moderate, 200″ ≤ RDS ≤ 400″ and C, good, RDS < 200″. Following the successful postoperative alignment, the proportion of patients with BiS were increased from 9.2 to 40.8%, 17.1 to 53.9%, 21.1 to 76.1% and 21.1 to 72.4% at 100%, 10%, 5% and 2.5% contrasts respectively. At 2.5% contrast, (1) more patients presented binocular summation in the groups B and C; (2) postoperative improvements of binocular visual acuity (BVA) in groups B (1.5 ± 1.03 lines) and C (1.57 ± 1.26 lines) were significantly different from the BVA in the group A (0.74 ± 1.00 line); and (3) in the group with central fusion, more patients presented BiS after surgery and the postoperative BVA improved by 1.43 ± 1.16 lines. Binocular summation for high contrast and different low contrasts can be improved in patients with IXT after successful surgical treatment. The improvement of BiS was associated with obtaining central fusion, recovering distant stereopsis and good alignment after the surgeries. The most significant improvement was shown at 2.5% contrast and was associated with good stereopsis and central fusion. The improvement of BiS, particularly at low contrast, has benefits for the daily activities in the real environment. BiS improvement could be used as a supplementary assessment of binocular function in patients with IXT before and after treatment.

## Introduction

Intermittent exotropia (IXT) is characterized by occasional divergent misalignment of one of both eyes^1^. In IXT, the binocular vision is destroyed gradually at distance and then at near^2,3^. Traditionally, clinicians test patients using Bagolini lenses, Worth-4-dot test, Titmus and random dots stereograph (RDS) to evaluate the binocular vision function. These tests introduce an artificial situation that may affect the test results to a greater or lesser degree^4^.

Binocular summation is defined as the superiority of visual acuity when using binocular vision compared with monocular vision. Naturally, binocular visual acuity (BVA) is important for daily life activities. However, binocular summation is generally neglected in the clinical examinations for strabismic patients. It is affected by the age, interocular difference of monocular vision and the presence of strabismus^5^. In strabismus patients, the mean BiS for the low contrast decreases significantly and is eventually inhibited^5^. Previous studies have shown that impaired BiS at 1.25% contrast relates with diminished quality of life in patients with strabismus^6^. Improved BiS is thought to be a functional benefit from the surgical correction of strabismus conditions. According to the findings of a previous study, the proportion of strabismic patients with binocular summation was improved from 21 to 30% at 2.5% contrast and from 13 to 26% at 1.25% contrast after surgery^7^. Moreover, the number of patients with binocular inhibition (impaired BiS) decreased both in high and low contrast levels after the surgery^7^. In another study that involved various subtypes of strabismus, it was reported that esotropia have a lower effect on the post-surgical improvement of BiS, as compared with exotropia^7^. Binocular summation has not been adequately investigated in terms of binocular function (BVA) in patients with IXT before and after surgeries. Our study aimed to explore the BiS improvement in IXT following the successful postoperative alignment and its correlation with the traditional examinations of binocular vision.

## Subjects and methods

### Patients

This study conformed to the declaration requirement of Helsinki for research involving human subjects. The protocol and waiver of informed consent used in this study were approved by Institutional Review Board (IRB) of Tianjin Eye Hospital (TJEH).

Seventy-six patients with IXT, underwent strabismus surgeries from October 2017 to April 2019. The patients attained successful postoperative alignment surgeries which were defined as the postoperative deviations ranging between 4 PD esophoria and 10 PD exotropia in the primary position both at near and at distance.

There were 30 female and 46 male patients, aged 9–40 years (16.7 ± 5.2 years on average). The preoperative exodeviations ranged from 20 to 65 PD (mean 41.8 ± 15.6 PD) at near and from 20 to 60 PD (mean 38.5 ± 13.5 PD) at distance. Before surgery, all the patients poorly controlled the alignment at distance (5 score based on Holmes control scale at distance) but could control alignment at near. All the patients lacked distant stereopsis tested by random dots stereograph (RDS) at 3 m and showed suppression tested by Worth-4-dot test at distance before the surgeries. The study excluded the patients with amblyopia, anisometropia (asymmetric astigmatism > 1.0 D and asymmetric spherical lens > 1.5 D), nystagmus, vertical deviation, dissociated deviation, mental mal-developmental and neurological abnormalities. None of the participants had previous strabismus surgery.

### Examinations

All the patients were examined by the same ophthalmologist. Prism and alternative cover test were used to measure deviations in the primary position and in 9-gaze. The assessments of binocular vision were taken while patients was wearing spectacles with full refraction correction. The conducted tests included Bagolini lens test, Worth-4-dot test (standard flashlight projecting at 3 m for central fusion), Titmus test and distant Random Dots Stereograph (P/N 1006, Vision assessment Corporation, Illinois, USA).

High contrast (100%) and Low contrast (10, 5, and 2.5%) visual acuities were tested using LEA numbers acuity tests (Part B courtesy Good-Lite Company, Streamwood, and IL.) at 3 m in a dim room. All the patients underwent binocular visual acuity (BVA) and monocular visual acuity tests in the different contrasts during preoperative and postoperative visits. The scores of visual acuity (log Mar) was recorded when patients was recognizing the full line. We calculated BiS from the differences between BVA and monocular visual acuity. Binocular summation (BiS) score was classified into three situations: (1) BiS > 1, BVA better than that of the better eye > 1 line, called binocular summation; (2) BiS < − 1, BVA worse than that of the better eye > 1 line, called binocular inhibition; (3) − 1 ≤ BiS ≤ 1, called equality. The results of distant RDS were grouped into: A, nil, unable to recognize; B for moderate, 200″ ≤ RDS ≤ 400″ and C for good, RDS < 200″. The results of Worth-4-dot test at distance were central suppression, central fusion and diplopia.

### Statistical analysis

The postoperative BiS scores in different groups were compared using one-way ANOVA and *t* test. Fisher’s exact post hoc tests were performed for pairwise comparisons of proportions in preoperative and postoperative patients with different BiS. All data were analyzed using SPSS, version 20.0 (IBM, Armonk, NY, USA). The significant difference between the different BiS were reported at P ˂ 0.05 for preoperative and postoperative patients.

### Ethics approval

The Institutional Review Board of Tianjin Eye Hospital approved this retrospective study of the medical records of the patients.

## Results

### Preoperative and postoperative BiS for different contrasts in patients with IXT

There were significant differences between preoperative and postoperative proportions of the patients with different BiS for all contrasts (Fisher’s exact test, P = 0.00 at 100% contrast; P = 0.00 at 10% contrast; P = 0.00 at 5% contrast and P = 0.00 at 2.5% contrast) (Table 1.) After surgeries, the number of the patients with BiS increased from 9.2 to 40.8% at 100% contrast, from 17.1 to 53.9% at 10% contrast, from 21.1 to 76.1% at 5% contrast and from 21.0 to 72.4% at 2.5% contrast. At the same time, the proportions of the patients with binocular inhibition (impaired BiS) at the four tested contrasts decreased.

**Table 1:** The numbers and proportions of the patients with different BiS Scores before and after strabismic Surgeries.

	100% contrast visual acuity			10% contrast visual acuity			5% contrast visual acuity			2.5% contrast visual acuity		
	BiS > 1 n (%)	− 1 ≤ BiS ≤ 1 n (%)	BiS < − 1 n (%)	BiS > 1 n (%)	− 1 ≤ BiS ≤ 1 n (%)	BiS < − 1 n (%)	BiS > 1 n (%)	− 1 ≤ BiS ≤ 1 n (%)	BiS < − 1 n (%)	BiS > 1 n (%)	− 1 ≤ BiS ≤ 1 n (%)	BiS < − 1 n (%)
Preoperative	7 (9.2%)	60 (78.9%)	9 (11.8%)	13 (17.1%)	57 (75.0%)	6 (7.9%)	16 (21.1%)	54 (71.1%)	6 (7.9%)	16 (21.0%)	54 (71.1%)	6 (7.9%)
Postoperative	31 (40.8%)	42 (55.3%)	3 (3.9%)	41 (53.9%)	33 (43.4%)	2 (2.6%)	51 (76.1%)	21 (27.6%)	4 (5.3%)	55 (72.4%)	20 (26.3%)	1 (1.3%)
P value	0.00			0.00			0.00			0.00		

There were significant differences between preoperative proportions and postoperative proportions of the patients with different BiS for all contrasts (Fisher’s exact test).

*Pre-op* preoperative, *Post-op* postoperative, *n* number, *BiS* binocular summation.

The postoperative proportions of patients with different BiS also differed significantly at the four tested contrasts (Fisher’s exact test, P = 0.01). The pairwise comparative analysis show that the significant differences existed between the postoperative proportions of patients (with different BiS) at 100% contrast and that at 5% contrast (Fisher’s exact test, P = 0.022). Similarly, the postoperative proportions of patients with different BiS was statistically different at 100% contrast and that at 2.5% contrast (Fisher’s exact test, P = 0.001). Postoperative BiS more improved at low contrast than that at high contrast. However, there was no significant difference between improvement of BiS in other groups (P = 0.273 between 100 and 10% contrast, P = 0.096 between 10% and 5% contrast, P = 0.057 between 10% and 2.5% contrast, and P = 0.429 between 5% and 2.5% contrasts, respectively).

### Postoperative BiS in groups with different RDS at distance

As shown in Table 2, the number of the patients with different BiS score in the three groups was based on the results of postoperative distant RDS. The proportion of the patients with different BiS differed significantly at only 2.5% contrast among three groups (P = 0.001). But there was no significant difference in the proportion of the patients with different BiS at 100%, 10% or 5% contrasts (P = 0.661, 0.220, 0.079, respectively). Using the 2.5% contrast LEA visual chart, more patients presented binocular summation in group B with moderate RDS at distance (85.7%) and group C with good RDS (86.4%) than the patients in group A with nil RDS (46.1%).

**Table 2:** The numbers of the patients with different BiS Scores after strabismic Surgeries.

	100% contrast visual acuity			10% contrast visual acuity			5% contrast visual acuity			2.5% contrast visual acuity		
	BiS > 1 n	− 1 ≤ BiS ≤ 1 n	BiS < − 1 n	BiS > 1 n	− 1 ≤ BiS ≤ 1 n	BiS < − 1 n	BiS > 1 n	− 1 ≤ BiS ≤ 1 n	BiS < − 1 n	BiS > 1 n	− 1 ≤ BiS ≤ 1 n	BiS < − 1 n
Group A (n = 26)	8	17	1	12	14	0	13	12	1	12	14	0
Group B (n = 28)	14	13	1	16	12	0	21	6	1	24	4	0
Group C (n = 22)	9	12	1	13	7	2	17	3	2	19	2	1
P	0.661			0.220			0.079			0.001		

Depending the results of distant RDS: A, nil, unable to recognize; B for moderate, 200″ ≤ RDS ≤ 400″, C for good, RDS < 200″.

The proportion of the patients with different BiS at 2.5% contrast differed significantly among three groups (P = 0.001).

*n* number, *BiS* binocular summation.

**Figure 1:**
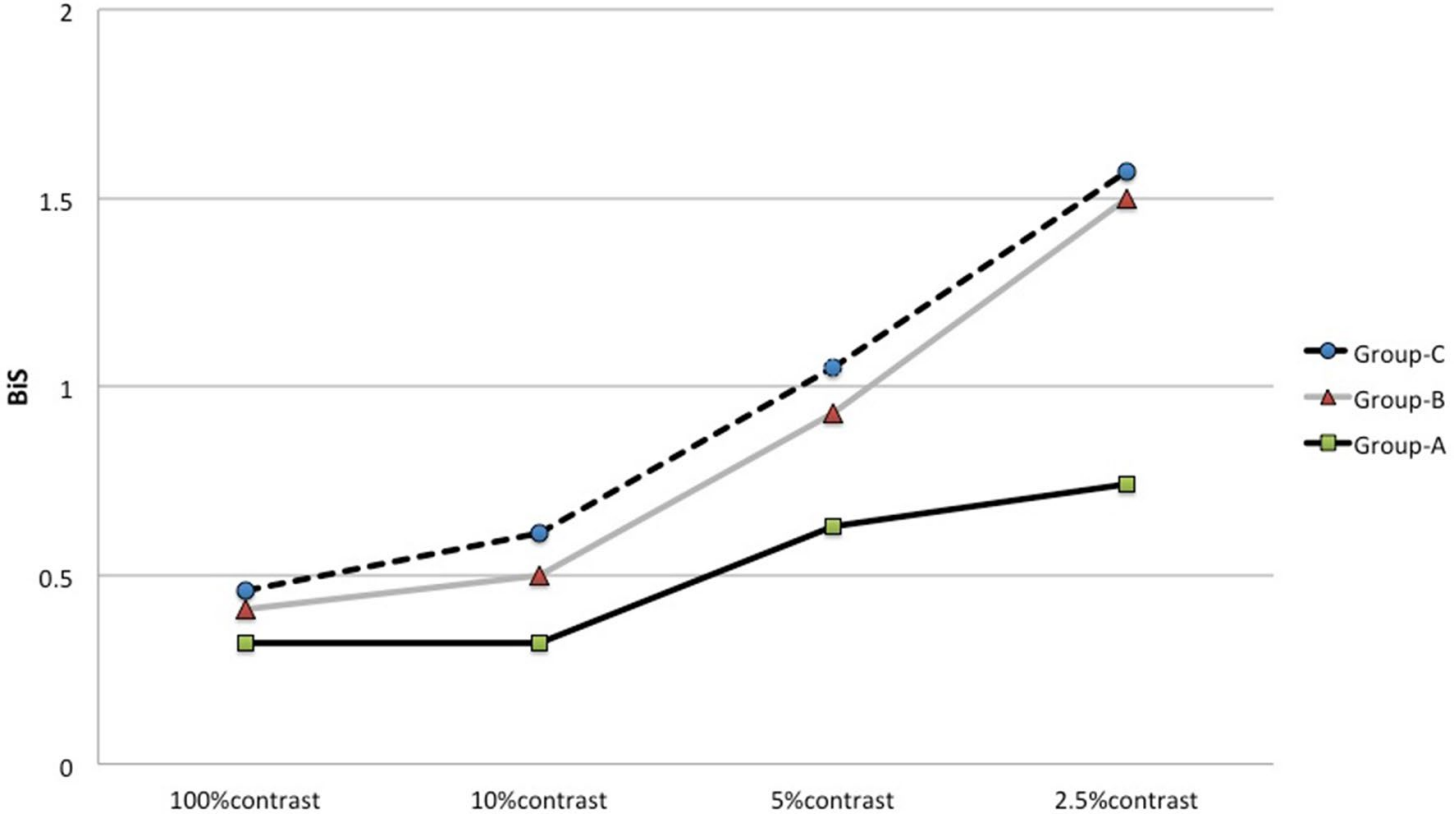
The average BiS records at different contrasts in three groups based on distant RDS after surgery.

Table 3 and Fig. 1 represent the average BiS records at high and low contrasts. According to the 2.5% contrast LEA visual chart, the postoperative BiS scores among the three groups were significantly different (P = 0.01). In average, the patients with moderate and good RDS at distance after surgery obtained binocular summation (1.5 lines and 1.57 lines, respectively) using 2.5% contrast LEA visual chart.

**Table 3:** The average BiS records (x ± s lines) for high contrast and different low contrast in three groups after strabismic surgeries.

	At 100% contrast	At 10% contrast	At 5% contrast	At 2.5% contrast
Group A	0.32 ± 0.68	0.32 ± 0.62	0.63 ± 1.10	0.74 ± 1.00
Group B	0.41 ± 0.57	0.5 ± 0.56	0.93 ± 0.81	1.5 ± 1.03
Group C	0.46 ± 0.66	0.61 ± 0.97	1.05 ± 0.95	1.57 ± 1.26
F	0.28	1.03	0.38	4.70
P	0.80	0.36	0.68	0.01*

*P < 0.05.

### Relationship of BiS score and Worth-4-dot test

**Figure 2:**
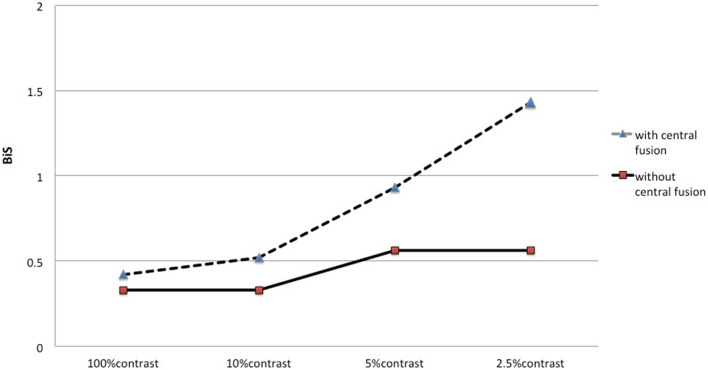
The average BiS records for different contrasts in the groups with central fusion and suppression.

The results of Worth-4-dot test showed that 15 patients had central suppression and 61 obtained binocularly central fusion after the surgeries. The numbers of patients with different BiS and the average BiS records for high and low contrasts were showed in Tables 4 and 5. There was significant difference only in the number of the patients with different BiS between the patients with central fusion and those with suppression at 2.5% contrast (P = 0.005). The number of patients with binocular summation at 2.5% contrast remarkable increased to 49 (80.3%) in patients with central fusion, compared with that in patients with suppression (6 cases, 40%). At 100%, 10% and 5% contrasts, the average BiS record was statistically similar in the patients with central fusion and those with suppression (P > 0.05). However, at 2.5% contrast the average BiS record was significant different between the patients with central fusion and those with suppression (P = 0.01). Furthermore, only using 2.5% contrast LEA visual chart, the patients with central fusion showed binocular summation 1.43 lines in average (Fig. 2).

**Table 4:** The numbers of the patients with different BiS Scores in the groups with and without central fusion after strabismic surgeries.

	100% contrast visual acuity			10% contrast visual acuity			5% contrast visual acuity			2.5% contrast visual acuity		
	BiS > 1	− 1 ≤ BiS ≤ 1	BiS < − 1	BiS > 1	− 1 ≤ BiS ≤ 1	BiS < − 1	BiS > 1	− 1 ≤ BiS ≤ 1	BiS < − 1	BiS > 1	− 1 ≤ BiS ≤ 1	BiS < − 1
Suppression (n = 15)	3	11	1	8	7	0	8	6	1	6	9	0
Central fusion (n = 61)	28	31	2	32	27	2	43	15	3	49	11	1
P	0.164			1.000			0.392			0.005		

*n* number, *BiS* binocular summation.

**Table 5:** The average BiS records (x ± s lines) for high and different low contrasts in patients with and without central fusion after strabismic surgeries.

	At 100% contrast	At 10% contrast	At 5% contrast	At 2.5% contrast
Group A	0.42 ± 0.62	0.52 ± 0.60	0.93 ± 0.88	1.43 ± 1.16
Group B	0.33 ± 0.38	0.33 ± 0.67	0.56 ± 0.92	0.56 ± 0.88
Group C	0.05	0.05	0.05	6.75
F	0.82	0.82	0.83	0.01*

*P < 0.05.

## Discussion

Binocular summation is the improvement in visual acuity when using binocular vision compared with the visual acuity of monocular vision. Binocular summation (BiS) was calculated as a ratio between the binocular score and the better-eye score, which was close to the estimated 1.4 in normal subjects in laboratory measures^8^. On average, the visual acuity of binocular vision at high contrast improved to 7%, while BiS at low contrast improved to approximately 40% or more in normal participants^9^. A 10% contrast is suggested as a more sensitive measure of both monocular and binocular visual function in multiple sclerosis and retrobulbar neuritis, associated with the brain MRI lesion burden^10^. Reductions in vision-specific quality of life were associated with lower scores at the low contrast^10,11^. In children, 65.7% had binocular summation, 28.6% had binocular equivalency and 5.7% had binocular inhibition^12^. The age, visual acuity of the better eye, interocular difference of monocular vision and stereoacuity affects BiS^5,11,13^.

Generally, binocular interaction is affected by the presence of strabismus. The BiS was more sensitive at the low contrast than that at the high contrast (100%). Pineles et al. reported that the mean BiS at 2.5% and 1.25% contrasts were lower in the strabismic patients than in control group. However, there was no significant difference in BiS at 100% contrast. The mean BiS of 0.9 at 1.25% contrast in strabismic patients demonstrated binocular inhibition and was associated with decreased quality of life^6^. Anika reported an average BiS score of − 2.14 letters at 1.25% contrast in the strabismic patients. This explain the blur vision complained by strabismic patients that cannot be demonstrated by the routine examinations^6^. Jaffer et al., reported that although the BiS at high contrasts was not associated with stereoacuity, the BiS at 2.5% contrast was significantly related to both near and distance stereoacuity^14^. Melinda reported that subnormal BiS at contrast of 1.25% in strabismic amblyopes was similar to that in strabismic patients preoperatively. Strabismus surgery also improved low-contrast BiS in strabismic amblyopes^15^. The number of patients with BiS was increased from 21 to 30% at 2.5% contrast and from 13 to 26% at 1.25% contrast after surgeries. Meanwhile, the number of patients with binocular inhibition was decreased after surgeries at all the contrast levels^7^.

Although stereoacuity and BiS for low contrast are correlated, they might be independently affected by strabismus. Having measurable preoperative stereoacuity and older onset of strabismus were the main factors of improved BiS after the surgeries^15^. In these previous studies, the enrolled subjects had all subtypes of strabismus. Binocular summation (BiS) might be more affected in esotropia than in exotropia^16,17^. Patients with infantile esotropia and childhood-onset strabismus had deeper suppression than patients with other subtypes so that they had less improved BiS scores after strabismus surgery^7^. Patients with IXT could maintain some degree of binocular visual function even before surgery. Stereoacuity at distance was variable in 42% patients^18^. Deterioration in near stereoacuity at 1–2 years follow-up is infrequent in IXT^19^. Chang reported binocular inhibition shown in 39.2% of patients with IXT for 100% contrast. Distance BVA correlated with a decreased distance stereoacuity but not with the size of the deviation. It might be helpful to assess the deterioration of alignment in IXT^15^. Jeong found that binocular CS summation ratio was correlated with the control scale in IXT and could be a useful method of assessing binocular visual function in IXT^20^. We supposed BiS in IXT have more improvement than that in other types of strabismus after surgery. However, improved BiS at the high and low contrasts in IXT, has not been investigated adequately in the previous study as the functional benefit from the surgical treatments.

In this study, it was evident that the proportions of patients with binocular summation were significantly increased for all the contrasts (100, 10, 5 and 2.5%) postoperatively. More patients attained binocular summation at 5% contrast and 2.5% contrast after surgeries. We considered that our results of surgical effect on BiS were better than those in the previous studies because only the patients with IXT were enrolled. Furthermore, this study excluding other subtypes of strabismus, amblyopia, anisometropia, nystagmus, and mental maldevelopment. The postoperative improvement of BVA at contrast of 2.5% differed significantly among the three groups of the patients with different distant RDS and between the groups with and without central fusion. We suggest that BiS at 2.5% contrast might be associated with binocular vision and might be as a predictor of attaining stereopsis.

In this study, some patients without stereopsis or central fusion also presented binocular summation. Viewing objects at 10° eccentric to the fovea was found to reduce VA by about 8 ETDRS lines. This showed that strabismus caused an image to fall into the extra fovea (non-corresponding retinal location) in the deviated eye. This may have led to interocular difference and therefore decrease in BiS, or even caused binocular inhibition^6,13^. Therefore, we suggest that BiS can be improved in patients without stereopsis or central fusion by proper alignment after strabismus surgeries. Furthermore, although the improvement was most evident at low contrasts, it was also observed at high contrast in some patients with good alignment after surgery.

Our study has several limitations. The study did not analyze the relationship between BiS and the result of Bagolini lens tests after surgery. This was because there were only four patients with suppression using Bagonili lens after surgery. The relationship between BiS and the near stereopsis (tested by Titmus) was not investigated. All the patients had good outcomes after surgeries because the postoperative examinations were taken at 2–3 month after surgery. Further studies are recommended to investigate the long-term improvement of BiS.

In conclusion, the BiS at high contrast (100%) and different low contrasts (10, 5 and 2.5%) can be improved in patients with IXT after surgeries. The improvement could be associated with obtaining central fusion, recovering stereopsis at distance and good alignment after surgeries. Improvement of BiS in patients with IXT, particularly at 2.5% contrast, benefits daily activities in the real environment and enhances the quality of life. To evaluate improvement of vision function in the real environment, we suggest that the BiS can be used as a supplementary assessment of binocular function in the patients with and without stereopsis and central fusion before and after surgeries.
